# Prevalence and comparative risk of mental health disorders in persons with vitiligo: a retrospective matched cohort study using claims data with expert-informed case validation

**DOI:** 10.1136/bmjopen-2025-106687

**Published:** 2026-03-04

**Authors:** Theresa Klinger, Matthias Augustin, Gregor Leicht, Steffen Moritz, Kristina Hagenström

**Affiliations:** 1 Institute for Health Services Research in Dermatology and Nursing (IVDP), University Medical Center Hamburg-Eppendorf, Hamburg, Germany; 2 Department of Psychiatry and Psychotherapy, University Medical Center Hamburg-Eppendorf, Hamburg, Germany

**Keywords:** prevalence, statutory health insurance, routine data, chronic skin disease, psychological diseases

## Abstract

**Objectives:**

To estimate the prevalence and comorbidity patterns of mental health disorders (MHDs) in persons with vitiligo and compare relative risks (RRs) with dermatological diseases (atopic dermatitis and psoriasis) and non-vitiligo comparator cohorts using claims data.

**Design:**

Retrospective matched cohort study using nationwide statutory health insurance claims data.

**Setting:**

German statutory health insurance claims (DAK-Gesundheit), 2016–2020.

**Participants:**

A 40% anonymised sample of insured persons (N=2 885 984). In 2020, persons with vitiligo (International Statistical Classification of Diseases, Tenth Revision, German Modification L80) were compared with cohorts with atopic dermatitis (L20), psoriasis (L40) and persons without vitiligo. Cohorts were propensity score matched (1:3) by age and sex.

**Primary outcome measures:**

Prevalence of MHDs and RRs across comparator cohorts.

**Secondary outcome measures:**

Internal plausibility of expert-informed claims-based MHD case definitions using diagnosis-only versus diagnosis-plus-care criteria.

**Results:**

In 2020, 4 631 persons were diagnosed with vitiligo. Affective disorders and neurotic, stress-related and somatoform disorders showed the highest prevalence, with depressive episodes ranging from 8.9% to 19.2% and somatoform disorders from 5.3% to 17.9% across definitions. In matched comparisons with atopic dermatitis, only a few and inconsistent differences were observed. In contrast, more pronounced and consistent differences were identified in comparisons with psoriasis. Emotional disorders in childhood showed higher risks under the most sensitive case definition (RR=2.29, 95% CI 1.14 to 4.61), whereas hyperkinetic disorders showed consistent effects across all definitions (RR range=1.58–1.93). Compared with persons without vitiligo, risks were higher for social phobia (RR range=2.10–2.81) and anxiety disorders (RR range=1.64–1.93).

**Conclusions:**

Persons with vitiligo show a substantial burden of affective and stress-related MHDs. The mental health comorbidity profile was largely comparable to that of atopic dermatitis, whereas more pronounced differences were observed in comparisons with psoriasis. Claims-based prevalence estimates were sensitive to case definition, highlighting the importance of transparent operationalisation.

**STRENGTHS AND LIMITATIONS OF THIS STUDY**

- The study is based on a large, population-based sample of German statutory health insurance data, enabling stable prevalence estimates and matched cohort comparisons with minimal selection bias.
- Mental health disorders were operationalised using expert-informed claims-based case definitions that combined International Statistical Classification of Diseases, Tenth Revision, German Modification diagnoses with care-related markers (medications and psychotherapy procedures).
- Propensity score matching was applied to construct comparable cohorts for vitiligo, atopic dermatitis, psoriasis and individuals without vitiligo based on age and sex.
- Claims data are collected for reimbursement purposes and may be affected by misclassification, incomplete outpatient coding and absence of inpatient medication data.
- Claims data do not capture mental health conditions not leading to healthcare utilisation, which may result in the underestimation of true prevalence.

## Introduction

 Vitiligo is a multifactorial autoimmune skin disease characterised by immune-mediated destruction of melanocytes, with well-documented associations to autoimmune dysregulation, genetic susceptibility and environmental triggers.^1^,^4^ Treatment options aim to improve the appearance of the skin, although a cure remains elusive.^5 6^ Vitiligo is a significant health problem with an estimated prevalence in the general population ranging from approximately 0.06% to 2.28%, whereas substantially higher prevalence rates have been reported in selected regional and clinical populations.^7^,^9^ Persons with vitiligo and those with other chronic skin conditions such as atopic dermatitis (AD) or psoriasis often suffer from various comorbidities. Numerous published studies also point to an increased risk of mental health disorders (MHDs) such as depression, insomnia, anxiety, stress and adjustment disorders, especially in persons with vitiligo and the entire group of people with chronic skin conditions.^9^,^13^ Studies have also reported a significant reduction in quality of life due to vitiligo, which can lead to anxiety, depression and even suicidal tendencies.^14 15^ Stigmatisation (eg, nasty comments and bullying) is also one of the most serious psychosomatic stresses for persons with vitiligo.^11^ However, these studies do not focus on the representativeness of MHDs in Statutory Health Insurance (SHI) data,^9^ but look at selected MHDs without validating them. Such an analysis would be a good compromise for prevalence estimates compared with other cross-sectional populations.^16^ A major challenge in studying vitiligo-related MHDs within SHI data is the validity of diagnoses. SHI data are primarily collected for billing rather than for epidemiological research, leading to potential inconsistencies in International Statistical Classification of Diseases, Tenth Revision (ICD-10), German Modification (GM) coding. Misclassification, incomplete coding and the differentiation between past and active diagnoses can distort prevalence estimates.^14^ A systematic internal validity study is necessary to evaluate the consistency and plausibility of ICD-10-coded MHD diagnoses in persons with vitiligo. This assessment is essential for ensuring the accuracy of prevalence estimates and identifying potential biases in administrative healthcare data.

The primary aim of this study was to estimate the prevalence and comorbidity patterns of MHDs in persons with vitiligo and compare relative risks (RRs) with cohorts of persons with AD, psoriasis and individuals without vitiligo using SHI claims data.

The secondary aim was to develop and internally assess expert-informed, claims-based case definitions for MHDs. As no external clinical reference standard is available in routine claims data, internal validation was conceptualised as an assessment of plausibility and internal consistency based on an expert consensus classification of ICD-10 GM diagnoses, psychotherapeutic procedures and psychotropic medications and on the systematic evaluation of alternative case definitions with increasing diagnostic specificity.

## Methods

### Study design, data source, and population

This retrospective health service study is based on an anonymised 40% sample of routine data from the German health insurance company DAK-Gesundheit (DAK-G) (n=2 885 984, 56.8% women, average age 48.6 years) of all insured persons who were insured for at least 1 day between 2016 and 2020.

### Consensus process and identification of mental health disorder care–related parameters and case definitions

As part of a multi-stage expert consensus process involving two psychiatrists and one dermatologist, diseases and care-related parameters relevant for mapping MHDs in routine SHI data were identified. The consensus process followed a structured, iterative approach. First, the expert panel compiled a preliminary selection of potentially relevant ICD-10 GM diagnosis codes, psychotherapeutic services and psychotropic medication independently. These proposals were then discussed in moderated consensus rounds in the second step. They were evaluated on the basis of clinical plausibility, relevance to care and expected specificity for MHDs. They were finalised by majority vote.

The expert panel then classified the eye validity of the identified parameters according to their specificity for MHDs, with 1 indicating high specificity, 2 indicating medium specificity and 3 indicating low specificity. As routine data lacks any external clinical reference standards, the validation approach was designed as an internal consistency and plausibility check of SHI-based case definitions. Based on the expert classification, three alternative annual case definitions with increasing diagnostic stringency were developed, representing different positions along a sensitivity–specificity continuum (case definition 1, ≥1 MHD diagnosis; case definition 2, ≥1 MHD diagnosis, medication or therapy; case definition 3, ≥1 MHD diagnosis and specific medication with eye validity 1).

### Identification of persons with vitiligo, atopic dermatitis and psoriasis

To determine the number of insured persons with vitiligo (ICD-10 GM L80) and the comparison cohorts of persons with other chronic skin diseases like AD (ICD-10 GM L20) and psoriasis (ICD-10 GM L40), as well as persons without vitiligo in Germany, diagnoses from 2016 to 2020 were taken into account. Persons with an outpatient diagnosis and/or a primary or secondary diagnosis during an inpatient stay in 2020 were included. The ‘without vitiligo’ cohort (defined as individuals without an ICD-10 GM L80 diagnosis in 2020) was established as a population-based reference group. This group was not restricted to individuals who were otherwise healthy to reflect the actual morbidity structure of the population covered by SHI and avoid selection bias resulting from the use of artificially healthy control groups. The study population was continuously insured (at least 1 day per quarter) during the year of observation. Individuals who died during the observation period were not excluded from the analyses.

### Statistics

Annual prevalence and incidence rates were expressed as percentages with corresponding 95% Wilson CIs for the years of observation, which were determined taking age and sex into account.^17 18^ The numerator of the estimator was the number of insured persons in the exposed cohort. The denominator consisted of all continuously insured persons in the sample. To generate comparable cohorts, nearest neighbour propensity score (PS) matching (PSM) was performed at a ratio of 1:3 based on age and sex. The selection of matching variables was deliberately limited to age and sex, as these are the strongest and most readily available confounders of both dermatological diseases and mental morbidity. The matching model was not extended to include comorbidity indices, utilisation indicators or socioeconomic proxies due to high missing rates, risk of over-adjustment (eg, for disease burden) and potential selection and collider effects. Residual confounding influences are considered a limitation. Minor case-wise deletion was applied because missingness affected core identification variables (ICD-10 GM diagnosis codes, age or sex) required for cohort definition and PSM, which cannot be validly imputed without introducing substantial bias.

Logistic regression was used to generate the required PS. RRs and 95% CI represented differences between the comparison cohorts. All analyses were performed with SAS V.9.4 (SAS Institute Inc., Cary, North Carolina [NC], USA).

### Patient and public involvement

Patients and public were not involved in the design and conduct of the study, the choice of outcome measures and recruitment of the study.

## Results

### Consensus process and eye validity

Following the consensus process, 21.25% of all available three-digit ICD-10 GM MHD codes were excluded (online supplemental table S1), whereas the remaining codes included in the analysis were rated with eye validity 3. Of a total of 12 care-related medications identified, 10 were rated with eye validity 1, and two others were rated with eye validity 2 and 3 due to their specificity for MHDs. Psychotherapeutic interventions were given apparent validity 3 because, despite their effectiveness, the lack of rigorous evidence, unclear treatment guidelines and possible neglect of individual experience in diagnosis may affect validity (online supplemental table S2).^19 20^ Based on the two consensus procedures and the categorisation of the inclusion criteria by visual validation, all three case definitions were then established (table 1).

**Table 1:** Case definition and eye validity to identify persons with vitiligo and mental health disorders in statutory health insurance data

Group (ident- ification)	Parameter		Eye validity	Case definition														
				1	2							3						
				F00–F99	F00 to F09	F10 to F19	F20 to F39	F40 to F49/ F60 to F69	F50 to F59	F70 to F79	F90 to F99	F00 to F09	F10 to F19	F20 to F39	F40 to F49/ F60 to F69	F50 to F59	F70 to F79	F90 to F99
Diagnosis (ICD-10)	(F-codes		3	x	x	x	x	x	x	x	x	x	x	x	x	x	x	x
		AND																
Drug (ATC)	Antidepressants)	OR	1		x		x	x				x		x	x			
	Antipsychotics/neuroleptics	OR	1		x		x	x	x	x		x		x	x	x	x	
	Mood stabiliser	OR	1		x		x			x		x		x			x	
	Anti-dementia	OR	1		x							x						
	Acamprosate	OR	1			x							x					
	Buprenorphine	OR	1			x							x					
	Clomethiazole	OR	1			x							x					
	Naltrexone	OR	1			x							x					
	Atomoxetine	OR	1								x							x
	Methylphenidate	OR	1								x							x
	Anxiolytics	OR	2		x	x	x											
	Hypnotics	OR	3				x	x	x									
		OR																
Therapy (EBM)	(online supplemental table S1)		3		x	x	x	x	x	x	x							

NOTE: 1, base definition; 2, extendet definition; 3, strict definition.

ATC, Anatomical-Therapeutic-Chemical Classification System (Anatomisch-Therapeutisch-ChemischesKlassifikationssystem); EBM, Uniform Assessment Standard (Einheitlicher Bewertungsmaßstab); ICD-10 GM, International Classification of Diseases, 10th Revision, German Modification.

### Mental health in persons with vitiligo

In 2020, 4631 persons (mean age 57 years, 2919 females) were diagnosed with vitiligo as their main or secondary diagnosis in an inpatient or outpatient setting. This corresponds to a prevalence of 0.19% (CI 0.19 to 0.20, ~1 48 437 persons in Germany, ~ 83 943 females).

The groups ‘affective disorders’ and ‘neurotic, stress-related and somatoform disorders’ had the highest prevalence rates across all case definitions. With prevalence rates of 19.15% (95% CI 18.02 to 20.29, case definition 1), 14.79% (95% CI 13.77 to 15.81, case definition 2) and 8.85% (95% CI 8.04 to 9.67, case definition 3), depressive episodes were the most common MHDs, corresponding to between 28 378 and 61 393 persons in Germany. Somatoform disorders were also among the five most common MHDs, with prevalence rates ranging from 17.90% (95% CI 16.80 to 19.01, case definition 1) to 5.27% (95% CI 4.63 to 5.91, case definition 3). Some MHDs, such as adjustment disorders, showed large decreases in prevalence between case definitions, whereas others, such as recurrent depressive disorders, showed only small changes. Mental disorders caused by tobacco use had a prevalence of 6.33% in case definition 1, which decreased to 0% in case definition 3.

**Figure 1:**
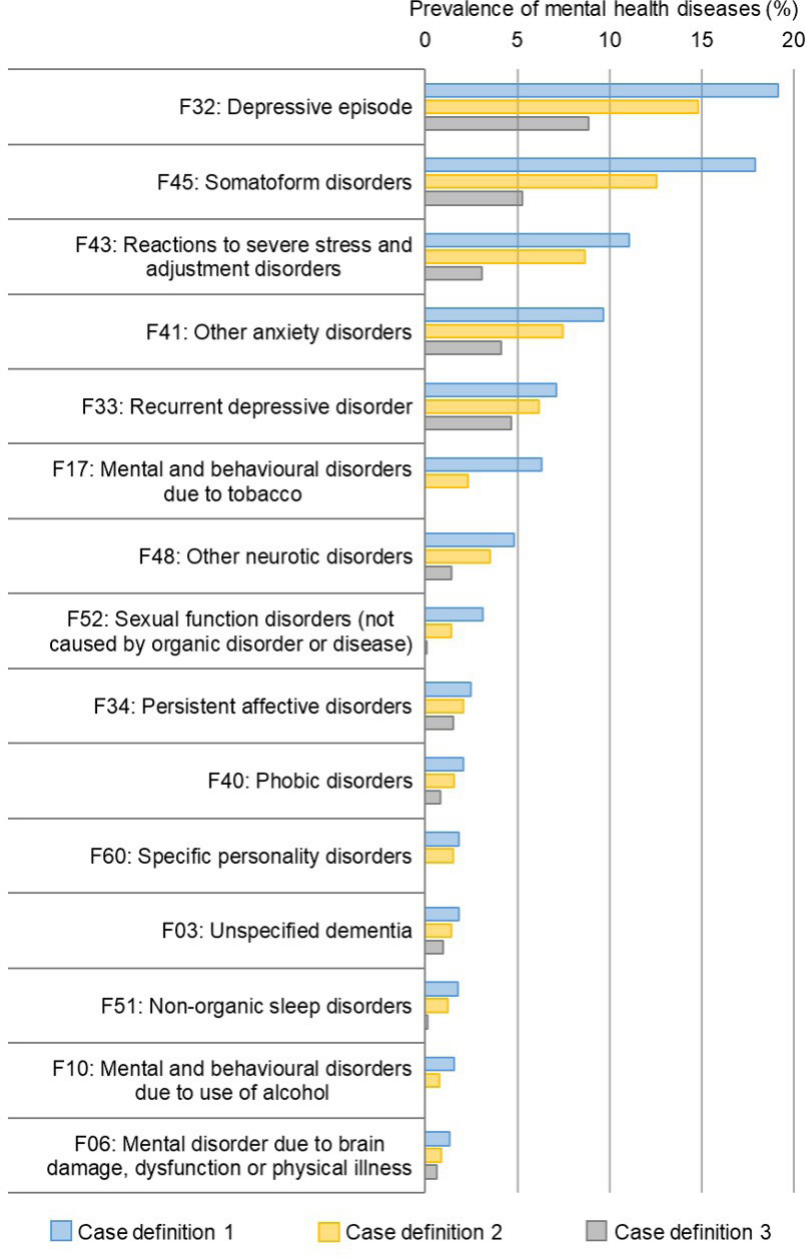
Prevalence of top 15 three-digit ICD-10 GM mental health codes of persons with vitiligo (ICD-10 L80, n=4631) according to different case definitions. *Note: case definition 1, ≥1 MHD ICD-10* GM *diagnosis; case definition 2, ≥1 MHD ICD-10* GM *diagnosis, medication or therapy; case definition 3, ≥1 MHD ICD-10* GM *diagnosis and specific medication.* ICD-10 GM, International Classification of Diseases, 10th Revision, German Modification; MHD, mental health disorder.

#### Comparisons to persons with atopic dermatitis and psoriasis

**Figure 2:**
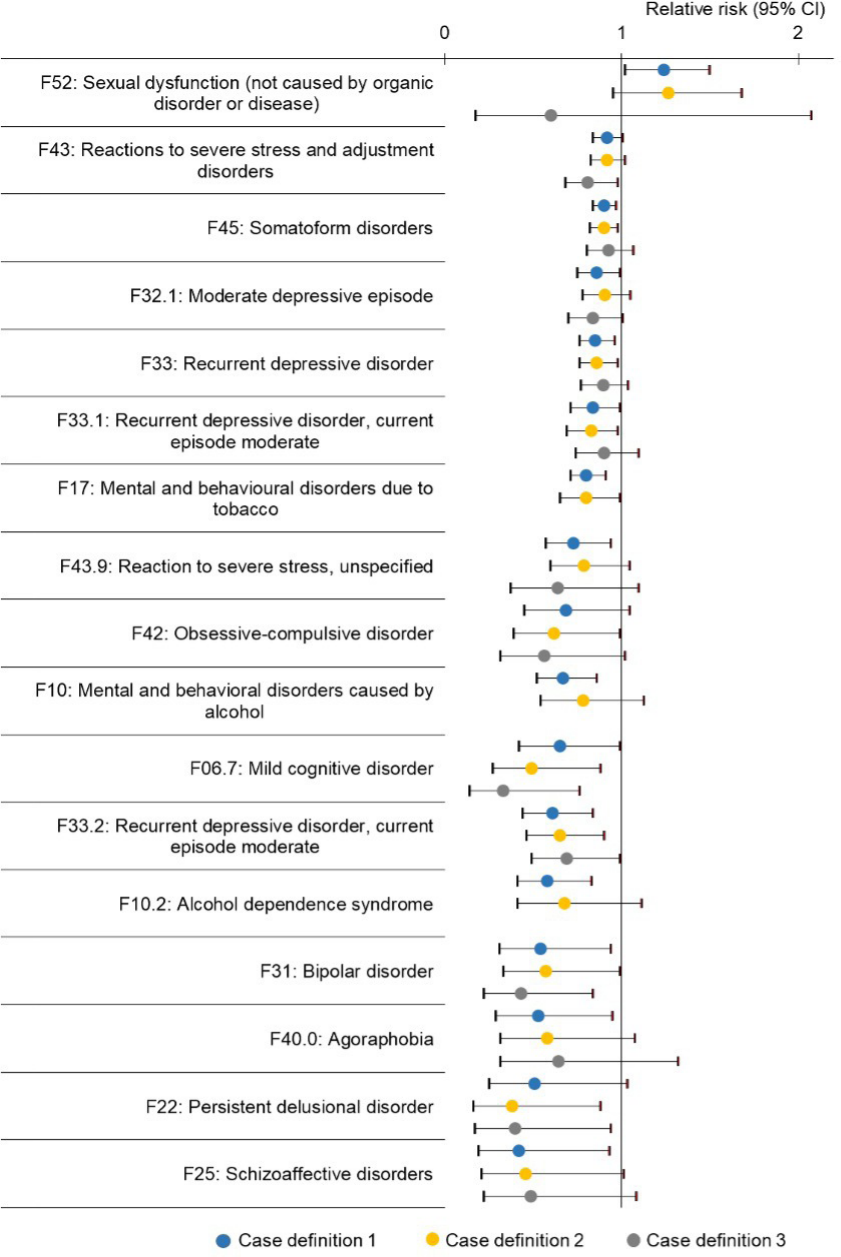
RR for mental disorders in persons with vitiligo (ICD-10 GM L80, n=4631) compared with persons with atopic dermatitis (ICD-10 GM L20, n=13 893) according to all three case definitions (data adjusted by propensity score matching 1:3 for age and sex). ICD-10 GM, International Classification of Diseases, 10th Revision, German Modification; RR, relative risk.

Compared with persons with AD (case definition 1), persons with vitiligo had a 24% higher risk of sexual dysfunction (95% CI 1.02 to 1.50) and a 58% lower risk of schizoaffective disorders (RR=0.42, 95% CI 0.19 to 0.93). No significant associations were observed for case definitions 2 and 3. Some MHDs showed protective effects only under stricter diagnostic criteria—for instance, persistent delusional disorder was associated with a lower risk in case definition 2 (RR=0.38, 95% CI 0.16 to 0.88) and case definition 3 (RR=0.40, 95% CI 0.17 to 0.94). For certain conditions, the protective effect persisted across all definitions. Persons with vitiligo consistently showed a lower RR of mild cognitive disorder (case definitions 1 to 3: RR range=0.33–0.65) and recurrent depressive disorder, current episode moderate (RR range=0.61–0.69; figure 2).

Persons with psoriasis had more significant differences in several MHDs to persons with vitiligo than persons with AD. The highest RR for case definition 1 was observed for obsessive–compulsive personality disorder (RR=2.36, 95% CI 1.38 to 4.03), with significant effects persisting in all case definitions. Persons with vitiligo also had a 129% higher risk of developing an emotional disorder in childhood (95% CI 1.14 to 4.61) than those with psoriasis, though this was not significant in case definitions 2 and 3. However, hyperkinetic disorders showed consistent effects after all case definitions (RR range=1.58–1.93). For social phobia and adjustment disorder, significant effects were found only in case definition 1 (RR=1.61, 95% CI 1.03 to 2.51; RR=1.16, 95% CI 1.01 to 1.32), with no effects after adding a supply parameter. Protective effects for persons with vitiligo were observed in mental and behavioural disorders due to psychotropic substances under case definitions 1 and 2, including dependence syndrome due to alcohol (RR range=0.44–0.47) and acute alcohol intoxication (RR range= 0.24–0.24). As in the comparison above, no effects were found for case definition 3 in this group of conditions.

**Figure 3:**
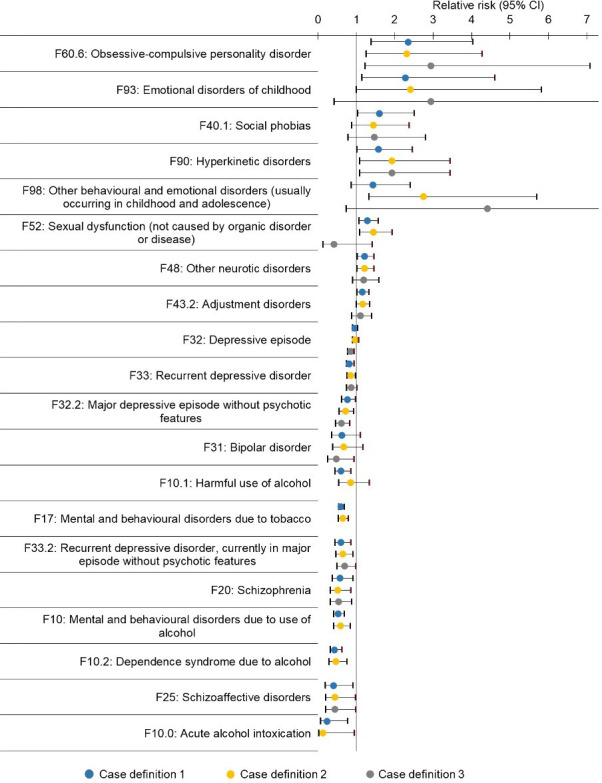
RR for mental disorders in persons with vitiligo (ICD-10 GM L80, n=4 631) compared with insured persons with psoriasis (ICD-10 GM L40, n=13 598) according to all three case definitions (data adjusted by propensity score matching 1:3 for age and sex). ICD-10 GM, International Statistical Classification of Diseases, Tenth Revision, German Modification; RR, relative risk.

#### Comparisons to persons without vitiligo

Many neurotic stress and somatoform disorders (F40–F49) also showed higher risks in persons with vitiligo, with social phobia having the strongest association (case definition 1, RR=2.81, 95% CI 1.71 to 4.62; case definition 2, RR=2.28, 95% CI 1.38 to 4.11; case definition 3, RR=2.10, 95% CI 1.06 to 4.15) followed by phobic disorders and anxiety disorders with significant RRs after all case definitions ranging from 1.64 to 1.93 across definitions. Although severe intellectual disability showed the highest RR under case definition 1 (RR=3.50, 95% CI 1.18 to 10.40), the wide CI indicates small subgroup sizes and possible chance effects, and the association was not significant when stricter case definitions were applied.

Combined and other personality disorders showed a high RR for case definition 1 (RR=2.40, 95% CI 1.12 to 5.12) but were no longer significant when an additional inclusion criterion was added in case definitions 2 and 3. Obsessive–compulsive disorder showed an increased risk for case definitions 1 and 2. Affective disorders (F30–F39) were consistently associated with a higher risk for vitiligo, with the exception of recurrent depressive disorder in remission and minor depressive episodes in case definition 3. In general, the effects in this comparison were much more consistent between the different case definitions.

**Figure 4:**
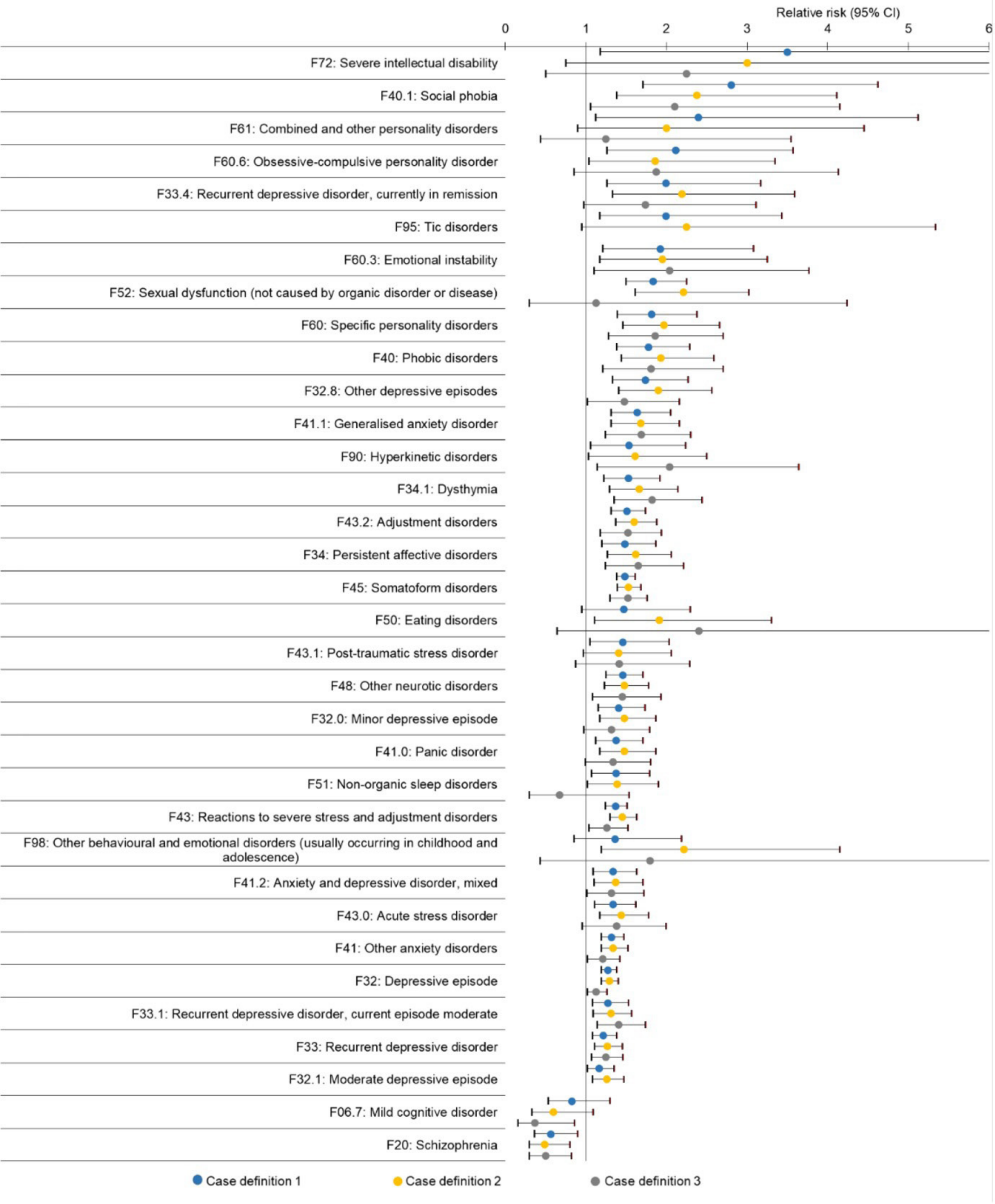
RR for mental disorders in persons with vitiligo (ICD-10 GM L80, n=4 631) compared with persons without vitiligo (n=13 893) according to all three case definitions (data adjusted by propensity score matching 1:3 for age and sex). ICD-10 GM, International Statistical Classification of Diseases, Tenth Revision, German Modification; RR, relative risk.

## Discussion

This study uses SHI data to estimate the prevalence and comorbidity patterns of MHDs in persons with vitiligo, comparing them with adjusted dermatological and non-vitiligo cohorts. Expert-developed case definitions were applied to perform an internal plausibility check across the sensitivity–specificity spectrum.

Affective disorders, particularly depressive episodes and recurrent depressive disorders, exhibited the highest prevalence across all case definitions, proving them to be diagnostically robust entities in routine data. The comparatively stable prevalence estimates for these disorders across various definitions suggest a high level of consistency in ICD-10 GM coding and associated care pathways. These prevalence rates were also consistent with the comorbidity rates reported in the literature for individuals with vitiligo.^1221^,^31^ Somatoform and stress-related disorders (F40–F49) exhibited relevant prevalence rates but showed greater declines when stricter case definitions were applied. This suggests that these disorders are more frequently coded in routine data based on diagnosis alone and are less frequently associated with specific therapeutic or pharmacological interventions. Case definition 3 was highly specific but less sensitive and is therefore more suitable for etiological analyses and risk estimation than for descriptive prevalence surveillance. It required an ICD-10 GM diagnosis in combination with specific psychotropic medication (eye validity 1). It probably primarily captures more severe cases that are clinically manifest and have been treated. However, it could underestimate disorders that are often not treated with medication in clinical practice, such as anxiety disorders or adjustment disorders. This should be considered alongside the high comorbidity of vitiligo with such disorders.^12 32^

Few statistically significant differences were observed between vitiligo and AD, indicating a largely comparable MHD comorbidity profile. In contrast, comparisons with psoriasis revealed more pronounced and consistent differences. Several associations observed under the most sensitive case definition became weaker as diagnostic stringency increased, suggesting possible overestimation under the least specific definition. This emphasises the descriptive–epidemiological nature of prevalence estimates. However, some MHDs from the group of behavioural and emotional disorders with onset in childhood and adolescence (F90–F99) showed detrimental effects for persons with vitiligo compared with persons with psoriasis. Because of its high visibility, vitiligo can be associated with social stigma, discrimination and psychological distress even in childhood and adolescence.^28^ Conversely, psoriasis can often be hidden by clothing. This visibility could lead to a comparatively higher psychosocial burden in persons with vitiligo compared with persons with psoriasis, even in early childhood.^33 34^ In addition, psychological distress in persons with psoriasis is often driven by the inflammatory nature of the disease, pain, itching and systemic comorbidities, rather than cosmetic appearance, which may shift psychological distress more towards coping with physical discomfort and less towards physical appearance. Comparisons between persons with vitiligo and persons without vitiligo showed consistent effects across all case definitions for depression and anxiety. Both MHDs are also described in the literature as relevant comorbidities.^13 35^ The impact of psychological distress in vitiligo is widely recognised. Studies show that visible skin changes contribute to increased social anxiety and avoidance behaviour and can lead to enormous stress, especially in persons with dark skin, due to increased visibility.^34 36^ In contrast, the isolated high effect observed for severe intellectual disability is most likely explained by selection effects and small subgroup sizes rather than a disease-specific association.

Overall, affective disorders and neurotic, stress-related and somatoform disorders were the most prevalent MHDs among persons with vitiligo. Prevalence estimates decreased systematically with increasing diagnostic stringency, which is more likely to be due to the internal validation strategy than to methodological inconsistencies. In matched comparisons, vitiligo’s comorbidity profile in relation to MHDs was largely comparable to that of AD. By contrast, significantly more consistent and more numerous differences were observed in comparisons with psoriasis, indicating a distinct comorbidity profile for this condition. These results demonstrate heterogeneous, comparison-dependent risk patterns and emphasise the importance of sensitivity analyses based on transparently reported case definitions when estimating psychiatric comorbidity using claims data.

### Strengths and limitations

The strength of the SHI data lies in its broad, representative sample, which covers 90% of the German population. This minimises selection bias and provides a reliable basis for estimating epidemiological measures and making group comparisons.^37 38^ This strength is further enhanced by the use of three comparator cohorts with different clinical reference profiles and by evaluating all associations across three increasingly stringent case definitions. This allows the internal validity and robustness of the findings to be assessed across alternative operationalisations. However, there are some limitations. First, only outpatient ATC codes and Uniform Assessment Standard (Einheitlicher Bewertungsmaßstab) codes (psychotherapeutic intervention) were included, as inpatient ATC codes are not recorded in SHI data. However, as inpatient ATC codes are known to be sufficiently validated, it can be assumed that their inclusion would not have changed the results significantly.^39^ In addition, the coding of ICD-10 GM codes can be inaccurate due to insufficient differential diagnoses.^40^ For example, Giersiepen et al.^39^ found that between 2001 and 2003, only 7.4% of diagnoses included corresponding voluntary indicators for safety checks.^39^ Since 2009, contract doctors have been required to indicate the degree of diagnostic certainty when billing diagnoses. Nevertheless, studies point to persistent weaknesses in coding quality.^41^ This suggests that considering outpatient care alone may distort the prevalence of MHDs according to case definition 1, as potentially incorrect or incompletely coded outpatient diagnoses are not reliably captured. This suggests that looking only at outpatient care may bias the prevalence of MHD according to case definition 1, as potentially incorrect or incompletely coded outpatient diagnoses are not reliably captured.^39^ To address this issue, future studies should perform validation checks through further sensitivity analyses and data linkage with primary data to test classification accuracy. Second, underreporting of untreated morbidity, as individuals with MHDs often underreport symptoms or fail to seek treatment, could result in the underestimation of prevalence within SHI data.^42^ This could be mitigated by incorporating additional data sources or patient surveys to capture unreported cases. Lastly, missing billing data for non-reimbursed services, such as alternative therapies, could also lead to underestimation. Expanding the dataset to include out-of-pocket payments or conducting interviews could help address this gap.^43^ In summary, while the study benefits from extensive SHI data, addressing these limitations through validation, supplementary data (eg, linkage with primary data), and more inclusive data collection would improve the accuracy of MHD prevalence estimates and psychosocial comparisons.

## Conclusion

This study provides internally validated prevalence estimates for MHDs in individuals with vitiligo, based on data from SHI in Germany, and demonstrates that patterns of mental comorbidity can differ significantly between chronic inflammatory skin diseases. Applying multiple case definitions with increasing diagnostic stringency enabled a structured assessment of diagnostic uncertainty, supporting the use of claim-based sensitivity analyses in psychiatric epidemiology. In matched comparisons, the mental comorbidity profile in vitiligo was largely comparable to that in AD. However, the comparison with psoriasis revealed a greater number of significant and consistent differences between multiple diagnostic groups. This suggests that psoriasis has a distinct comorbidity profile. These findings highlight the heterogeneity of psychosocial comorbidity in dermatological diseases and caution against generalising psychological distress across different clinical presentations.

## Supplementary material

10.1136/bmjopen-2025-106687 online supplemental file 1

## Data Availability

Data are available upon reasonable request.
