# Supplementary material for: Prevalence and comparative risk of mental health disorders in persons with vitiligo: a retrospective matched cohort study using claims data with expert-informed case validation
Source: BMJ Open. 2026 Mar 4;16(3):e106687. doi: 10.1136/bmjopen-2025-106687 (PMC12970095; doi:10.1136/bmjopen-2025-106687)
Supplement: online supplemental file 1 [file bmjopen-16-3-s001.docx]

**APPENDIX**

**Table S1:** Three-digit ICD-10 GM mental health disorder codes excluded from the analytical cohort

| **Diagnostic group** | **ICD-10 GM** | **Mental Health Disorder** |
| --- | --- | --- |
| Organic mental disorders | **F09** | Unspecified organic or symptomatic mental disorder |
| Schizophrenia, schizotypal & delusional disorders | **F28** | Other nonorganic psychotic disorders |
|  | **F29** | Unspecified nonorganic psychosis |
| Mood [affective] disorders | **F38** | Other mood [affective] disorders |
|  | **F39** | Unspecified mood [affective] disorder |
| Behavioural syndromes with physiological disturbances | **F54** | Psychological and behavioural factors associated with disorders or diseases classified elsewhere |
|  | **F59** | Unspecified behavioural syndromes associated with physiological disturbances and physical factors |
| Disorders of adult personality and behaviour | **F68** | Other disorders of adult personality and behaviour |
|  | **F69** | Unspecified disorder of adult personality and behaviour |
| Disorders of psychological development | **F80** | Specific developmental disorders of speech and language |
|  | **F81** | Specific developmental disorders of scholastic skills |
|  | **F82** | Specific developmental disorder of motor function |
|  | **F83** | Mixed specific developmental disorders |
|  | **F84** | Pervasive developmental disorders |
|  | **F88** | Other disorders of psychological development |
|  | **F89** | Unspecified disorder of psychological development |
| Behavioural & emotional disorders with onset in childhood/adolescence | **F96** | Other behavioural and emotional disorders with onset usually occurring in childhood and adolescence |
|  | **F97** | Other behavioural and emotional disorders with onset usually occurring in childhood and adolescence |
| Mental disorder without further specification | **F99** | Mental disorder without further specification |

NOTE: ICD-10 GM: International Classification of Diseases, 10th Revision, German Modification

**Table S2:** Relevant therapies of persons with mental health diseases

| **EBM number** | **Form of therapy** |
| --- | --- |
| 22220/23220 | Psychotherapeutic interview |
| 30810 | Initial prescription for sociotherapy |
| 30811 | Review of the indication for follow-up sociotherapy prescription |
| 30930 | Disease-specific neuropsychological diagnostics using test procedures |
| 30931 | Probationary session |
| 30932/30933 | Neuropsychological therapy (individual/group treatment) |
| 30934 | Creating a therapy plan |
| 30935 | Report in the event of therapy extension in individual cases |
| 35100 | Differential diagnostic clarification of psychosomatic illnesses |
| 35110 | Verbal intervention for psychosomatic illnesses |
| 35111/35112 | Exercise-based interventions, individual or group treatment |
| 35113 | Exercise-based interventions for children and adolescents, group treatment |
| 35120 | Hypnosis |
| 35130/35131 | Determination of the obligation to pay benefits for the initiation/extension of short-term therapy |
| 35140 | Biographical anamnesis |
| 35141 | In-depth exploration |
| 35142 | Supplementary assessment of neurological and psychiatric findings |
| 35150 | Probationary session |
| 35151 | Psychotherapeutic consultation hours |
| 35152 | Psychotherapeutic acute treatment |
| 35163 to 35169 | Complex for probationary sessions in a group setting (group treatment) |
| 35173 to 35179 | Complex for basic group psychotherapeutic care (group treatment) |
| 35200 to 35203 | Depth psychology-based psychotherapy (short-term therapy/long-term therapy, individual treatment/large group) |
| 35205/35208 | Depth psychology-based psychotherapy for children and adolescents (short-term therapy/long-term therapy, small group) |
| 35210/35211 | Analytical psychotherapy (individual treatment/large group) |
| 35212 | Analytical psychotherapy for children and adolescents (small group) |
| 35220 to 35225 | Behavioral therapy (short-term therapy/long-term therapy, individual treatment/small group/large group) |
| 35251 to 35253 | Surcharge I, II, III |
| 35300/35301 | Test procedures, standardized/psychometric |
| 35302 | Procedure, projective |
| 35401/35402/35405 | Depth psychological psychotherapy (STT 1/STT 2/LTT, individual treatment) |
| 35411/35412/35415 | Analytical psychotherapy (STT 1/STT 2/LTT, individual treatment) |
| 35421/35422/35425 | Behavioral therapy (STT 1/STT 2/LTT, individual treatment) |
| 35431/35432/35435 | Systemic therapy (STT 1/STT 2/LTT, individual treatment) |
| 3550X | Depth psychology-based psychotherapy (group treatment) (STT) |
| 3551X | Depth psychology-based psychotherapy (group treatment) (LTT) |
| 3552X/3553X | Analytical psychotherapy (STT/LTT) |
| 3554X/3555X | Behavioral therapy (STT/LTT) |
| 35571/35572/35573 | Supplement for individual therapy/group therapy/acute treatment |
| 35591 | Surcharge for STT, individual treatment |
| 35593 to 35599 | Surcharge for STT group, 3 to 9 participants |
| 35600/35601 | Test procedures, standardized/psychometric |
| 35602 | Procedure, projective |
| 37500 | Initial consultation |
| 37520 | Creation of an overall treatment plan in accordance with § 9 of the KSVPsych-RL |
| 37525 | Additional flat rate for services provided by the referring physician or psychotherapist |
| 37530 | Coordination of care in accordance with Section 10 of the KSVPsych-RL by a non-medical person in accordance with Section 5 (2) of the KSVPsych-RL |
| 37535 | Home visit |
| 37550 | Case discussion according to § 6 KSVPsych-RL |
| 37551 | Supplement to the fee schedule item 37550 |
| 37570 | Additional flat rate for additional organizational and management tasks as well as technical expenses within the framework of a network association |
| 88130/88131 | Termination of psychotherapy without/with subsequent relapse prophylaxis |
| 88220 | is added to the basic flat rate to indicate that only video sessions have taken place in the quarter |

NOTE: STT=short-term therapy; LTT=long-term therapy, EMB: Uniform assessment standard (Einheitlicher Bewertungsmaßstab)
