# Supplementary material for: Prevalence and comparative risk of mental health disorders in persons with vitiligo: a retrospective matched cohort study using claims data with expert-informed case validation
Source: BMJ Open. 2026 Mar 4;16(3):e106687. doi: 10.1136/bmjopen-2025-106687 (PMC12970095; doi:10.1136/bmjopen-2025-106687)
Supplement: Supplementary file 2 [file bmjopen-2025-106687.reviewer_comments.pdf]

PEER REVIEW HISTORY

BMJ Open publishes all reviews undertaken for accepted manuscripts. Reviewers are asked to complete a checklist review form and are provided with free text boxes to elaborate on their assessment. These free text comments are reproduced below.

ARTICLE DETAILS

Title (Provisional)

Prevalence and comparative risk of mental health disorders in persons with vitiligo: a retrospective matched cohort study using claims data with expert-informed case validation

Authors

Klinger, Theresa; Augustin, Matthias; Leicht, Gregor; Moritz, Steffen; Hagenström, Kristina

---

VERSION 1 - REVIEW

**Reviewer**	**1**
**Name**	**Ravenscroft, Jane**
**Affiliation**	**Nottingham University Hospitals NHS Trust, Department of Dermatology**
**Date**	**30-Sep-2025**
**COI**	**None**

This is an important piece of work looking at the mental health of a large number of patients.

I am unclear as to the main purpose and outcomes of the paper.

The title suggests validation of vitiligo and mental health disorders in claims data. Later the text says that this was not a diagnostic test procedure but rather a categorisation of information or services that could be classified on the basis of their apparent validity at first glance. I am not clear who/how the consensus process was carried out and what the subsequent case definitions mean, including their relative perceived accuracy or usefulness.

I think this section of the paper needs describing in detail so it is open to scrutiny. Perhaps it should be a separate paper.

The second part of the paper looks at relative prevalence of mental health disorders in different groups of patients. The variation in results depending on which mental health definition is used means it is difficult to interpret the data.

The chosen comparators ie atopic dermatitis, psoriasis or 'no vitiligo' are interesting to dermatologists. However, patients with 'no vitiligo' may have a range of other serious health conditions so it is difficult to draw any conclusions.

Only a very small number of results have been selected for mention in the results and conclusion section. I appreciate there is more data in the tables but these are difficult to understand. In particular, in the abstract results section, results are given re risk of mild cognitive disorder vs patients with psoriasis; and social phobia and anxiety vs those without vitiligo. In contrast the conclusion states the overall mental health burden of vitiligo and overall comparison with psoriasis.

Overall i feel the data needs presenting in a different way to make it understandable to the target audience.

**Reviewer**	**2**
**Name**	**Gardner, Jackie**
**Affiliation**	**Vitiligo Support International, Lynchburg, Virginia**
**Date**	**15-Dec-2025**
**COI**	**Jackie Gardner has served as a consultant for AbbVie, Avita Medical, Clinuvel Pharmaceuticals, Concert Pharmaceuticals, Incyte, Mitsubishi Tanabe Pharma Corporation, Pfizer and Teva Pharmaceuticals.**

This topic is critically underreported and misunderstood by the world at large. The impact on mental health by a disease that robs individuals of their identity and self-worth continues to be summarily dismissed under the cloak of “it won’t hurt you.”

Physicians and insurance companies denying the importance of acknowledgement and treatment of the emotional and psychological impact of vitiligo only further denigrates the patient. However, beyond that, even families and loved ones closest to the patient truly have no idea of the degree of impact. In fact, the more well-adjusted a person may seem, can disguise the hidden lengths to which they go to create that façade.

I look forward to this paper being published to establish valid, documentable, evidence of one of the greatest obstacles vitiligo patients have faced in their recovery.

Regarding the following 2 questions from the Review Checklist:

**7. If statistics are used are they appropriate and described fully?**	**no**
**8. Are the references up-to-date and appropriate?**	**no**

The following statement **(Page 6/30 Line 11)** is wildly inaccurate:

> “**Vitiligo is a significant health problem with a highly ranges worldwide estimated prevalence from 0.06 to 8.80%** [10–12].”

There are no sources citing "**worldwide**" vitiligo prevalence as high as 8.80%.

This is the source cited in the manuscript [10]

> 10. Krüger C, Schallreuter KU. A review of the worldwide prevalence of vitiligo in children/adolescents and adults. Int J Dermatol 2012;51(10):1206–12.doi:10.1111/j.1365- 4632.2011.05377.x [published Online First: 27 March 2012]. https://onlinelibrary.wiley.com/doi/abs/10.1111/j.1365-4632.2011.05377.x

Text below is taken from the above cited source:

> **Results** We found more than 50 studies that used several methods and subgroups of the general population. The prevalence of vitiligo ranges from 0.06 to 2.28%, whereas this was 0.0–2.16% in children/adolescents populations.
>
> **Conclusions** The often cited prevalence of 8% could not be confirmed after excluding clinical patient populations. **Accordingly, the worldwide prevalence of vitiligo ranges between 0.5 and 2%**.

They appear to be citing this paper:

> Vora RV, Patel BB, Chaudhary AH, Mehta MJ, Pilani AP. A Clinical Study of Vitiligo in a Rural Set up of Gujarat. Indian J Community Med. 2014 Jul;39(3):143-6. doi: 10.4103/0970-0218.137150. PMID: 25136154; PMCID: PMC4134529. https://pmc.ncbi.nlm.nih.gov/articles/PMC4134529/

In this paper, the 8.8% vitiligo prevalence statistic refers to the highest reported rates found in specific regions of India, particularly Gujarat and Rajasthan, not a "**worldwide**" average.

Consequently, this statement from the manuscript is inaccurate:

> **"Vitiligo is a significant health problem with a highly ranges worldwide estimated prevalence from 0.06 to 8.80% [10–12]**."

Next

I am not certain to which Review Checklist question/s they relate.

I marked "No" on the 2 questions below.

> **10. Are they presented clearly?**
> **11. Are the discussion and conclusions justified by the results**

The following statement **(Page 11/30 Line 10)** is incorrect:

> "**Severe intellectual disability had the highest RR for persons with vitiligo compared to those without**."

Vitiligo research shows associations with mental health disorders (depression, anxiety, stress, adjustment disorders), but not neurodevelopmental intellectual disability. Severe intellectual disability is not associated with vitiligo, and the **wording should be corrected to reflect relevant mental health conditions**.

Next

INTRODUCTION (page 6/30 Line 6)

“**Although an autoimmune reaction is suspected, the exact cause remains unclear** [6, 7].”

Yes, the etiology is complicated, however, the association to **autoimmunity is documented**, **not suspected**.

Both of the cited sources **(#6 and #7**) support autoimmunity

The first (**#6**) source is not necessarily related to the autoimmune reference. However, the discussion certainly confirms the association.

6. Böhm M, Schunter JA, Fritz K, et al. S1 Guideline: Diagnosis and therapy of vitiligo. J Dtsch Dermatol Ges 2022;20(3):365–78. doi:10.1111/ddg.14713 [published Online First: 4 March 2022]. https://onlinelibrary.wiley.com/doi/10.1111/ddg.14713

Two relevant sections from (#6) above source.

“NSV is associated with an increased rate of various autoimmune diseases, in particular thyroid diseases and alopecia areata [8]. For thyroid diseases and autoimmune thyroiditis, respectively”

“The closest association was found for circulating anti-thyroid peroxidase (anti-TPO) and anti-thyroglobulin
(anti-TG) antibodies. This is consistent with an increased prevalence of associated autoimmune thyroid disease (Hashimoto’s thyroiditis)”

#7 Source

7. Ezzedine K, Grimes PE, Meurant J-M, et al. Living with vitiligo: results from a national survey indicate differences between skin phototypes. Br J Dermatol 2015;173(2):607–09. doi:10.1111/bjd.13839 [published Online First: 28 July 2015]. https://jamanetwork.com/journals/jamadermatology/fullarticle/2785895

From the above (#7) Source:

“Introduction

“Vitiligo is an autoimmune disorder in which the immune system causes patchy loss of skin pigmentation.”

Additional independent sources documenting autoimmunity:

Upadhya, Sathvik et al. “Genetic and immune dysregulation in vitiligo: Insights into autoimmune mechanisms and disease pathogenesis.” Autoimmunity reviews vol. 24,8 (**2025**): 103841. doi:10.1016/j.autrev.2025.103841 https://www.sciencedirect.com/science/article/pii/S1568997225001016

“**Vitiligo disease pathogenesis involves autoimmune dysfunction, environmental factors, genetic**

predisposition, oxidative stress, metabolic dysfunction and environmental factors."

Chang, Wei-Ling et al. "Vitiligo: An Autoimmune Skin Disease and its Immunomodulatory Therapeutic Intervention." *Frontiers in cell and developmental biology* vol. 9 797026. 14 Dec. **2021**, doi:10.3389/fcell.2021.797026 https://pmc.ncbi.nlm.nih.gov/articles/PMC8712646/

"Vitiligo is a multifactorial disorder that combines genetic susceptibility, the generation of inflammatory mediators from environmental triggers, and autoimmune responses."

"Although many hypotheses have been proposed to explain the disease pathogenesis of vitiligo, autoimmune dysregulation theory is one of the widely accepted mechanisms."

Below is the text I entered in the "Comments to the Author"

This topic is critically underreported and misunderstood by the world at large. The impact on mental health by a disease that robs individuals of their identity and self-worth continues to be summarily dismissed under the cloak of "it won't hurt you."

Physicians and insurance companies denying the importance of acknowledgement and treatment of the emotional and psychological impact of vitiligo only further denigrates the patient. However, beyond that, even families and loved ones closest to the patient truly have no idea of the degree of impact. In fact, the more well- adjusted a person may seem, can disguise the hidden lengths to which they go to create that façade.

I look forward to this paper being published to establish valid, documentable, evidence of one of the greatest obstacles vitiligo patients have faced in their recovery.

I attached a document with comments and concerns for a couple of specifics.

**Reviewer**	**3**
**Name**	**Molenberghs, Geert**
**Affiliation**	**Universiteit Hasselt, Interuniversity Institute for Biostatistics and statistical Bioinformatics (I-BioStat)**
**Date**	**03-Jan-2026**
**COI**	**None**

The statistical design and analysis is generally adequate, although there is room for some improvement.

Propensity score matching is fine, but perhaps age and gender is a bit limited in terms of variables on which the matching is based. For example, what about comorbidities, socio-economic status, etc. This should be done or at least clearly commented on (if not available, and deemed appropriate, it could be added to the limitations).

Relative risks in terms of the reference groups are properly calculated and reported, including in the abstract and layman's summary. However, absolute quantities (prevalence)

are important as well. While reported in the manuscript itself, they should be part of, among others, the abstract.

Case-wise deletion has lost most of its credibility, especially in this day and age where sophisticated multiple imputation technology is available, e.g., based on FCS (full conditional specification).

VERSION 1 - AUTHOR RESPONSE

Reviewer 1 (Dr Jane Ravenscroft)

Comment R1.1: "I am unclear as to the main purpose and outcomes of the paper."

Response: We appreciate this point and have clarified the manuscript objectives and primary/secondary outcomes. We now explicitly distinguish (i) the comparative analysis of mental health outcomes across comparator groups using these definitions and (ii) the development/assessment of claims-based operational definitions ("internal diagnostic validation framework").

Change made: Objectives and outcomes clarified:

Abstract: *[…] Primary outcome measures: Prevalence of MHDs and relative risks (RRs) across comparator cohorts. Secondary outcome measures: Internal plausibility of expert-informed claims-based MHD case definitions using diagnosis-only versus diagnosis-plus-care criteria.*

Location: Abstract p. 2, l. 21–24

Introduction: *The primary aim of this study ~~research~~ was to estimate the prevalence and comorbidity patterns of MHDs in persons with vitiligo and to compare relative risks (RR) with cohorts of persons with atopic dermatitis, psoriasis, and individuals without vitiligo using SHI claims data. ~~investigate whether MHD can be precisely defined on the basis of coded diagnoses and additional information from SHI data.~~*

~~To clarify this, a consensus procedure involving clinicians and experts and a subsequent analysis of SHI data was carried out. The project also aimed to clarify which additional care-relevant information, apart from the diagnosis, could be considered in the SHI data to determine the prevalence of MHD and how it differs from that of other cohorts.~~

The secondary aim was to develop and internally assess expert-informed, claims-based case definitions for MHDs. As no external clinical reference standard is available in routine claims data, internal validation was conceptualised as an assessment of plausibility and internal consistency based on an expert consensus classification of ICD-10 diagnoses, psychotherapeutic procedures, and psychotropic medications, and on the systematic evaluation of alternative case definitions with increasing diagnostic specificity.

Location: Introduction p. 6, l. 30–32 and p. 7 l. 1-12

Comment R1.2: "The title suggests validation of vitiligo and mental health disorders in claims data. Later the text says that this was not a diagnostic test procedure but rather a categorisation of information or services that could be classified on the basis of their apparent validity at first glance."

Response A: We thank the reviewer for this important clarification. To avoid any misunderstanding regarding the nature of the validation procedure, we have revised the title to place the primary

focus on prevalence estimation and comparative risk assessment of mental health disorders in persons with vitiligo. The expert-informed case validation is now explicitly framed as a supporting methodological component rather than as a diagnostic test procedure.

Change made: Terminology standardised (e.g., “claims-based operational definitions” and “expert consensus-based classification of expected validity”) and the absence of an external reference standard is stated clearly.

Title: *Prevalence and comparative risk of mental health disorders in persons with vitiligo: a retrospective matched cohort study using claims data with expert-informed case validation* ~~*Internal diagnostic validation of vitiligo and mental health disorders in claims data*~~

SHORT-TITLE: *Prevalence of mental health disorders in vitiligo* ~~*Mental health of persons with vitiligo*~~

Location: Title p. 1, l. 1-6

Response B: In addition, we have revised both the Introduction and Methods sections to more clearly describe the approach as an internal assessment of claims-based operationalisations using an explicit expert consensus framework, rather than as an external validation against a clinical reference standard. The terminology has been harmonised throughout the manuscript to reflect this distinction consistently.

Introduction: ~~*To clarify this, a consensus procedure involving clinicians and experts and a subsequent analysis of SHI data was carried out. The project also aimed to clarify which additional care-relevant information, apart from the diagnosis, could be considered in the SHI data to determine the prevalence of MHD and how it differs from that of other cohorts.*~~ *[…] As no external clinical reference standard is available in routine claims data, internal validation was conceptualised as an assessment of plausibility and internal consistency based on an expert consensus classification of ICD-10 diagnoses, psychotherapeutic procedures, and psychotropic medications, and on the systematic evaluation of alternative case definitions with increasing diagnostic specificity.*

Location: Introduction p. 7, l. 3–12

Method - Consensus process and identification of *care-related parameters and case definitions: […] As routine data lacks any external clinical reference standards, the validation approach was designed as an internal consistency and plausibility check of SHI-based case definitions.*

Location: Methods p. 9, l. 30–32

Comment R1.3: “I am not clear who/how the consensus process was carried out and what the subsequent case definitions mean, including their relative perceived accuracy or usefulness. I think this section of the paper needs describing in detail so it is open to scrutiny.”

Response: We have expanded the methods section to provide a detailed description of the consensus process (including the composition of the panel, the contributions reviewed and exclusions in the supplemented Table S1, the contributions reviewed, the decision criteria and how disagreements are resolved). We have also clarified how each case definition should be interpreted (e.g. whether it is more specific or more sensitive) and its intended use (discussed e.g. for conservative epidemiological estimates or broad case capture). When no reference standard is available, we avoid quantifying diagnostic accuracy, but we do provide qualitative expectations and intended use cases.

Change made: Added a dedicated subsection describing the consensus procedure, including *two psychiatrists and one dermatologist,* materials reviewed: *one to identify relevant ICD-10-GM diagnosis codes, and another to identify psychotherapeutic services by EBM codes and psychotropic medications by ATC codes,* decision rules: *They were evaluated on the basis of clinical plausibility, relevance to care,*

and expected specificity for MHD, agreement/disagreement handling: *They were finalised by majority vote.,* added table s2 with excluded ICD-10 GM codes

Methods - Consensus process and identification of ~~*mental health disorders*~~ *care-related parameters and case definitions:* ~~*In a multi-stage consensus process involving experts (e.g. psychiatrists and psychologists), a majority decision was reached on which diseases and parameters should be considered relevant for the definition of MHD.*~~ *As part of a multi-stage expert consensus process involving two psychiatrists and one dermatologist, diseases and care-related parameters relevant for mapping MHD in routine SHI data were identified.* ~~*A total of two consensus processes was carried out, one identifying ICD-10 codes relevant to MHD and the other identifying treatments and medications relevant to MHD. Subsequently, specific and less specific inclusion criteria for the measurement of MHD were identified. This was not a diagnostic test procedure, but rather a categorisation of disease-specific information or services that could be classified on the basis of their apparent validity 'at first glance'.*~~ *The consensus process followed a structured, iterative approach. First, the expert panel compiled a preliminary selection of potentially relevant ICD-10-GM diagnosis codes, psychotherapeutic services, and psychotropic medication independently. These proposals were then discussed in moderated consensus rounds in the second step. They were evaluated on the basis of clinical plausibility, relevance to care, and expected specificity for MHD. They were finalised by majority vote.*

~~*As the proportion of misdiagnoses or preliminary diagnoses during a hospital stay is relatively high in the inpatient data, only the consensus ICD-10 codes were used as principal diagnoses to identify MHD. Treatments and drugs were identified using the uniform valuation standard (EBM) code or the anatomical-therapeutic-chemical classification system (ATC).*~~ *The expert panel then classified the eye validity of the identified parameters according to their specificity for MHD, with 1 indicating high specificity, 2 indicating medium specificity and 3 indicating low specificity. As routine data lacks any external clinical reference standards, the validation approach was designed as an internal consistency and plausibility check of SHI-based case definitions. Based on the expert classification, three alternative annual case definitions with increasing diagnostic stringency were developed, representing different positions along a sensitivity–specificity continuum (Case definition 1: ≥1 MHD diagnosis, Case definition 2: ≥1 MHD diagnosis, medication, or therapy, Case definition 3: ≥1 MHD diagnosis and specific medication wir eye validity 1).* ~~*Since the data structure does not allow a direct link between an ICD-10 code and another outpatient criterion, all parameters were recorded at the annual section level.*~~

Location: Methods, p. 8, l. 7–32 and p. 9, l. 1-6

Results: *Following the consensus process, 36% of all available three-digit ICD-10-GM MHD codes were excluded (Table S1), […]*

Location: Reults, p. 11, l. 5-6

Discussion: *Several associations observed under the most sensitive case definition became weaker as diagnostic stringency increased, suggesting possible overestimation under the least specific definition. This emphasises the descriptive-epidemiological nature of prevalence estimates.*

Location: Discussion, p. 15, l. 23–25

Discussion*: Case definition 3 was highly specific but less sensitive and is therefore more suitable for etiological analyses and risk estimation than for descriptive prevalence surveillance. It* ~~*which*~~ *required an ICD-10 diagnosis in combination with specific psychotropic medication (Eye validity 1). It probably primarily captures more severe cases that are clinically manifest and have been treated.*

Location: Discussion, p. 14, l. 33 and p. 15, l. 1–4

Comment R1.4: “The variation in results depending on which mental health definition is used means it is difficult to interpret the data.”

Response: We have revised the introductory section of the discussion to explicitly explain the conceptual framework and purpose of the three case definitions. We now clarify that, due to the lack of an external clinical reference standard in the claims data, the validation was designed as an internal plausibility and consistency assessment. The three case definitions were designed to represent different positions along a continuum of sensitivity and specificity, ranging from a sensitive surveillance definition to a highly specific definition that captures clinically manifest and treated MHD cases. We further explain that the observed decline in prevalence across case definitions reflects increasing diagnostic stringency and is a central element of the internal validation strategy, rather than a methodological inconsistency.

Change made: Added a detailed methodological interpretation of the prevalence gradients and their impact on diagnostic robustness in routine data.

Discussion: *Affective disorders, such as particularly depressive episodes and recurrent depressive disorders~~, were the most common (19.80% to 8.58% according to case definition 1 to 14.94% to 5.85% according to case definition 3)~~ exhibited the highest prevalence across all case definitions, proving them to be diagnostically robust entities in routine data. The comparatively stable prevalence estimates for these disorders across various definitions suggest a high level of consistency in ICD-10 coding and associated care pathways.*

Location: Discussion p. 14, l. 17–22

Conclusion: *Applying multiple case definitions with increasing diagnostic stringency enabled a structured assessment of diagnostic uncertainty, supporting the use of claim-based sensitivity analyses in psychiatric epidemiology.*

Location: Conclusion p. 18, l. 16–19

Comment R1.5: “However, patients with 'no vitiligo' may have a range of other serious health conditions so it is difficult to draw any conclusions.”

Response: We are aware that individuals in the 'without vitiligo' cohort may have other health issues. However, this group was defined as a population-based reference cohort rather than a healthy control group, in order to reflect the actual morbidity structure of the population covered by statutory health insurance, and to avoid selecting an artificially healthy comparison group. This design enables the relative psychological impact of vitiligo under routine care conditions to be estimated.

Methods - Identification of persons with vitiligo, atopic dermatitis, and psoriasis: *The 'without vitiligo' cohort (defined as individuals without an ICD-10 GM L80 diagnosis in 2020) was established as a population-based reference group. This group was not restricted to individuals who were otherwise healthy, in order to reflect the actual morbidity structure of the population covered by statutory health insurance, and to avoid selection bias resulting from the use of artificially healthy control groups.*

Location: Methods p. 9, l. 12–16

Comment R1.6: “Only a very small number of results have been selected for mention in the results and conclusion section. I appreciate there is more data in the tables but these are difficult to understand. In particular, in the abstract results section, results are given re risk of mild cognitive disorder vs patients with psoriasis; and social phobia and anxiety vs those without vitiligo. In contrast

the conclusion states the overall mental health burden of vitiligo and overall comparison with psoriasis.“

Response: We have comprehensively revised the discussion, results summary and conclusion sections to ensure consistent and transparent presentation of the central comparison patterns. The focus is now primarily on overarching result patterns of group comparisons and interpreting the case definitions and (e.g. vitiligo vs. AD, psoriasis and no vitiligo). Additionally, we report selected individual findings that were consistent across all case definitions (e.g. affective and anxiety disorders in vitiligo versus no vitiligo) or that are discussed in the literature as being clinically significant (e.g. disorders with an onset in childhood or adolescence, F90–F99, in vitiligo versus psoriasis). This allows for a coherent, clinically interpretable overall assessment.

Change made: The abstract, discussion and conclusions were harmonised, shifting the focus to consistent patterns of results and an interpretation of psychological comorbidity that is fully supported by the reported data.

Abstract: *Results: […] In matched comparisons with atopic dermatitis, only few and inconsistent differences were observed. […] Conclustion: Persons with vitiligo show a ~~significant mental health~~ substantial burden, ~~particularly for~~ of affective and stress-related MHDs~~disorders~~. ~~Compared to persons with psoriasis, persons with vitiligo showed less significant risks for some MHDs, suggesting greater psychological distress in the psoriasis cohort. Integrating mental health care into dermatological treatment is crucial, and further research is needed to refine case definitions and validate findings in longitudinal studies.~~ The mental health comorbidity profile was largely comparable to that of atopic dermatitis, whereas more pronounced differences were observed in comparisons with psoriasis. Claims-based prevalence estimates were sensitive to case definition, highlighting the importance of transparent operationalisation.*

Location: Abstract p. 2, l. 32–33 and p 3, l. 8-16;

Discussion*: ~~Comparisons between vitiligo and AD or psoriasis revealed mixed protective and harmful effects. Significant associations diminished with stricter case definitions, suggesting possible overestimation in definition 1. For example, sexual dysfunction associated with vitiligo was significant in case definition 1 but not in definitions 2 and 3. Similarly, disorders such as social phobias, emotional disorders of childhood, and adjustment disorders followed a comparable trend. A comparison of persons with vitiligo and persons with psoriasis showed a similar pattern of MHD with protective effects.~~ Few statistically significant differences were observed between vitiligo and atopic dermatitis, indicating a largely comparable MHD comorbidity profile. In contrast, comparisons with psoriasis revealed more pronounced and consistent differences. Several associations observed under the most sensitive case definition became weaker as diagnostic stringency increased, suggesting possible overestimation under the least specific definition. This emphasises the descriptive-epidemiological nature of prevalence estimates.*

Location: Discussion p. 15, l. 14–25;

Discussion (Summary)*: ~~In general, these results highlight the need for mental health interventions as part of dermatological care, particularly for affective and somatoform disorders. Given the increasing prevalence of depression in recent years [43]. continuous refinement of case definitions is essential to capture the evolving nature of MHD epidemiology. Furthermore, despite their significant impact on quality of life, somatoform disorders remain under-diagnosed and under-treated, suggesting a need for more integrative screening approaches in dermatology and mental health settings.~~ Overall, affective disorders and neurotic, stress-related and somatoform disorders were the most prevalent MHD among persons with vitiligo. Prevalence estimates decreased systematically with increasing diagnostic stringency, which is more likely to be due to the internal validation strategy than to methodological inconsistencies. In matched comparisons, vitiligo's comorbidity profile in relation to MHD was largely comparable to that of AD. By contrast, significantly more consistent and more*

numerous differences were observed in comparisons with psoriasis, indicating a distinct comorbidity profile for this condition.~~Furthermore, our results emphasize the need for sensitivity tests to refine prevalence estimates and to validate case definitions for MHD in individuals with vitiligo based on claims data.~~These results demonstrate heterogeneous, comparison-dependent risk patterns and emphasize the importance of sensitivity analyses based on transparently reported case definitions when estimating psychiatric comorbidity using claims data.

Location: Discussion p. 16 (summary), l. 12–29;

Conclusion: *This study provides ~~valuable insights into the mental health burden associated~~ internally validated prevalence estimates for MHD in individuals with vitiligo, based on data from SHI in Germany, and demonstrates that patterns of mental comorbidity can differ significantly between chronic inflammatory skin diseases. ~~and its comparison to individuals with AD, psoriasis, and those without vitiligo. The results demonstrate a significant prevalence of MHDs in individuals with vitiligo, particularly affective disorders, somatoform disorders, and stress-related conditions. By applying different case definitions and ensuring internal validity, the study provides a more accurate estimation of MHD prevalence. When comparing vitiligo patients with those having AD, psoriasis, or no vitiligo, important differences were observed in the comorbidity patterns of MHDs. Comparing persons with vitiligo and psoriasis, the latter had more significant harmful effects in MHD and all case definition than in the comparison of vitiligo and AD. Psoriasis is associated with more severe physical symptoms and a higher treatment burden, which increases psychosocial distress and the risk of psychological comorbidities. Persons with vitiligo also show high psychosocial burden and often severe disease progression, as significant risks were identified in all case definitions.~~ Applying multiple case definitions with increasing diagnostic stringency enabled a structured assessment of diagnostic uncertainty, supporting the use of claim-based sensitivity analyses in psychiatric epidemiology. In matched comparisons, the mental comorbidity profile in vitiligo was largely comparable to that in AD. However, the comparison with psoriasis revealed a greater number of significant and consistent differences between multiple diagnostic groups. This suggests that psoriasis has a distinct comorbidity profile. ~~The study's internal validity was strengthened by a multi-step consensus process and refined case definitions, reducing biases from misclassification and incomplete coding. By incorporating treatment and medication data, the study ensured a more accurate estimation of MHD prevalence beyond ICD-10-based diagnoses. This methodological rigor enhances the reliability of findings and underscores the importance of internal validity controls in epidemiological research. Future studies can build on this approach to improve the precision of MHD prevalence assessments in dermatological conditions, ultimately informing better-targeted mental health interventions.~~ These findings highlight the heterogeneity of psychosocial comorbidity in dermatological diseases and caution against generalising psychological distress across different clinical presentations.*

Location: Conclusion p. 18, l. 1–30

Comment R1.7: "Overall i feel the data needs presenting in a different way to make it understandable to the target audience."

Response: We would like to thank the reviewer for this valuable feedback. In order to make the results more comprehensible to the target audience, we have fundamentally revised the way they are presented. We have added a new overview table in which the three case definitions are presented transparently and classified along a sensitivity-specificity continuum. Additionally, the discussion section has been restructured to focus on key comparative patterns between vitiligo, atopic dermatitis, psoriasis, and individuals without vitiligo. Individual findings are now only

discussed as examples if they are consistent across all case definitions or are considered particularly clinically relevant in the literature. These changes make the presentation of the results much easier for a readership interested in clinical practice and healthcare science to understand.

Change made: Added a new overview table of the case definitions (sensitivity–specificity continuum) and restructured the Discussion to focus on the main comparative patterns.

Location: Table 1 p. 20; Discussion text p. 14-19;

==

Reviewer 2 (Dr Jackie Gardner; attached comments)

Comment R2.1 (Checklist Q7/Q8): "7. If statistics are used are they appropriate and described fully? No"

Response: We thank the reviewer for this valuable feedback. We have revised the section on methods to make the analyses clearer, more transparent and more reproducible. In particular, we now describe in more detail the consensus process in which we created the case definitions. We have also clarified why we used age- and sex-based propensity score matching as part of the internal validation strategy.

Change made: Identification of the case definition

Methods: ***Consensus process and identification of ~~mental health disorders~~ care-related parameters and case definitions:*** *~~In a multi-stage consensus process involving experts (e.g. psychiatrists and psychologists), a majority decision was reached on which diseases and parameters should be considered relevant for the definition of MHD.~~ As part of a multi-stage expert consensus process involving two psychiatrists and one dermatologist, diseases and care-related parameters relevant for mapping MHD in routine SHI data were identified. ~~A total of two consensus processes was carried out, one identifying ICD-10 codes relevant to MHD and the other identifying treatments and medications relevant to MHD. Subsequently, specific and less specific inclusion criteria for the measurement of MHD were identified. This was not a diagnostic test procedure, but rather a categorisation of disease-specific information or services that could be classified on the basis of their apparent validity 'at first glance'.~~ The consensus process followed a structured, iterative approach. First, the expert panel compiled a preliminary selection of potentially relevant ICD-10-GM diagnosis codes, psychotherapeutic services, and psychotropic medication independently. These proposals were then discussed in moderated consensus rounds in the second step. They were evaluated on the basis of clinical plausibility, relevance to care, and expected specificity for MHD. They were finalised by majority vote. ~~As the proportion of misdiagnoses or preliminary diagnoses during a hospital stay is relatively high in the inpatient data, only the consensus ICD-10 codes were used as principal diagnoses to identify MHD. Treatments and drugs were identified using the uniform valuation standard (EBM) code or the anatomical-therapeutic-chemical classification system (ATC).~~ Two independent consensus procedures were carried out for this purpose: one to identify relevant ICD-10-GM diagnosis codes, and another to identify psychotherapeutic services by EBM codes and psychotropic medications by ATC codes. The expert panel then classified the eye validity of the identified parameters according to their specificity for MHD, with 1 indicating high specificity, 2 indicating medium specificity and 3 indicating low specificity. As routine data lacks any external clinical reference standards, the validation approach was designed as an internal consistency and plausibility check of SHI-based case definitions. Based on the expert classification, three alternative annual case definitions with increasing diagnostic stringency were developed, representing different positions along a sensitivity–specificity continuum (Case definition 1: ≥1 MHD diagnosis, Case definition 2: ≥1 MHD diagnosis, medication, or therapy, Case definition 3: ≥1 MHD diagnosis and specific medication wir eye validity 1). ~~Since the data structure does not allow a direct link between an ICD-10 code and another outpatient criterion, all parameters were recorded at the annual section level.~~*

Location: Methods— Consensus process and identification of care-related parameters and case definitions p. 8, l. 7–32 and p. 9, l. 1-6

Change made: Describing the matching strategy and variables

Methods: *The selection of matching variables was deliberately limited to age and gender, as these are the strongest and most readily available confounders of both dermatological diseases and mental morbidity. The matching model was not extended to include comorbidity indices, utilisation indicators or socioeconomic proxies due to high missing rates, the risk of over-adjustment (e.g. for disease burden), and potential selection and collider effects. Residual confounding influences are considered a limitation.*

Location: Methods— Statistics p. 9, l. 27–33

Comment R2.2: "8. Are the references up-to-date and appropriate? No […] Prevalence statement inaccurate: worldwide … 0.06 to 8.80%"

Response: We thank the reviewer for pointing this out. We corrected the prevalence statement and revised the citations accordingly. The manuscript no longer reports 8.8% as a worldwide estimate; instead, we now report prevalence ranges supported by population-based evidence and clarify that higher values refer to selected regional/clinical samples rather than global prevalence.

Change made: Replaced the inaccurate wording and revised citations accordingly.

Introduction: *Vitiligo is a significant health problem with ~~a highly ranges worldwide estimated prevalence from 0.06 to 8.80%~~ an estimated prevalence in the general population ranging from approximately 0.06% to 2.28%, while substantially higher prevalence rates have been reported in selected regional and clinical populations [10–12].*

Location: Introduction p. 6, l. 7–11;

Comment R2.3: "The following statement (Page 11/30 Line 10) is incorrect:"Severe intellectual disability had the highest RR for persons with vitiligo compared to those´without."

Response: We have revised the wording to avoid implying a causal or clinically established association between vitiligo and severe intellectual disability. Although a high relative risk was observed under the most sensitive case definition (RR = 3.50, 95% CI 1.18–10.40), the very wide confidence interval indicates small subgroup sizes and potential random variation, and the association was no longer significant under stricter definitions. The result is now reported with appropriate caution and without overinterpretation. Furthermore, we have added a sentence to the Discussion clarifying that the isolated high relative risk observed for severe intellectual disability is most likely explained by selection effects and small subgroup sizes rather than a disease-specific association.

Change made: Results narrative revised; outcome handling clarified

Results: *Although severe intellectual disability showed the highest relative risk under case definition 1 (RR = 3.50, 95% CI 1.18–10.40), the wide confidence interval indicates small subgroup sizes and possible chance effects, and the association was not significant when stricter case definitions were applied.*

Location: Results - Comparisons to persons without vitiligo p. 13, l. 4–7;

Discussion: *In contrast, the isolated high effect observed for severe intellectual disability is most likely explained by selection effects and small subgroup sizes rather than a disease-specific association.*

Location: Discussion p. 16, l. 9–11;

Comment R2.4: "Although an autoimmune reaction is suspected, the exact cause remains unclear [6, 7]. Yes, the etiology is complicated, however, the association to autoimmunity is documented, not suspected."

Response: We thank the reviewer for this important clarification. We have revised the statement accordingly to reflect the well-established autoimmune aetiology of vitiligo and removed the wording suggesting uncertainty. The sentence has been reformulated to describe vitiligo as a multifactorial autoimmune skin disease with immune-mediated destruction of melanocytes. The additional references kindly provided by the reviewer were very helpful and have been incorporated into the revised manuscript.

Change made: Replaced "suspected" phrasing and updated supporting references

Introduction: *Vitiligo is a ~~skin condition in which melanocytes are destroyed, resulting in depigmented patches of skin. Although an autoimmune reaction is suspected, the exact cause remains unclear~~ [6, ~~7].~~ multifactorial autoimmune skin disease characterised by immune-mediated destruction of melanocytes, with well-documented associations to autoimmune dysregulation, genetic susceptibility, and environmental triggers [6, 7, 46, 47].*

Location: Introduction p. 6, l. 2–6;

===
=========

Reviewer 3 (Dr Geert Molenberghs)

Comment R3.1: „Propensity score matching is fine, but perhaps age and gender is a bit limited in terms of variables on which the matching is based. For example, what about comorbidities, socio-economic status, etc. This should be done or at least clearly commented on (if not available, and deemed appropriate, it could be added to the limitations)."

Response: Matching was restricted to age and sex because these variables are fully observed, stable over time, and represent the major confounders for both dermatological and mental health conditions. Additional variables such as comorbidity indices, healthcare utilisation proxies, or socioeconomic indicators (e.g. occupation-based activity codes as a proxy for SES) were considered but not included due to substantial missingness (>70% for the activity code), risk of overadjustment, and potential collider bias. Given the extent and structure of missing data, multiple imputation was deemed inappropriate and likely to introduce additional bias without meaningful gain in validity. Residual confounding is therefore acknowledged as a limitation. To strengthen internal validity and robustness of inference despite these constraints, we analysed three comparator cohorts with different clinical and epidemiological validity profiles, allowing triangulation of effect estimates across heterogeneous reference populations.

Change made: Discussed further confounding adjustment for PSM, highlight different group comparisons and case definitions as a strength.

Methods: *[…] The selection of matching variables was deliberately limited to age and gender, as these are the strongest and most readily available confounders of both dermatological diseases and mental*

morbidity. The matching model was not extended to include comorbidity indices, utilisation indicators or socioeconomic proxies due to high missing rates, the risk of over-adjustment (e.g. for disease burden), and potential selection and collider effects. Residual confounding influences are considered a limitation.

Location: Methods— Statistics p. 9, l. 28–33

Strength and Limitation: *[…] This strength is further enhanced by the use of three comparator cohorts with different clinical reference profiles, and by evaluating all associations across three increasingly stringent case definitions. This allows the internal validity and robustness of the findings to be assessed across alternative operationalization's. […]*

Location: Strengths and limitations p. 17, l. 3–7;

Comment R3.2: "However, absolute quantities (prevalence) are important as well. While reported in the manuscript itself, they should be part of, among others, the abstract."

Response: We agree and added absolute prevalence estimates for the key outcomes alongside relative measures in the abstract.

Change made: Abstract now includes prevalence (%).

Abstract: *Results: In 2020, 4,631 persons were diagnosed with vitiligo. Affective disorders and neurotic, stress-related and somatoform disorders showed the highest prevalence, with depressive episodes ranging from 8.9% to 19.2% and somatoform disorders from 5.3% to 17.9% across definitions. […]*

Location: Abstract - Results p. 2, l. 29–34;

Comment R3.3: „Case-wise deletion has lost most of its credibility, especially in this day and age where sophisticated multiple imputation technology is available, e.g., based on FCS (full conditional specification)."

Response: We thank the reviewer for this important methodological comment. In our study, missingness affected core identification variables (ICD-10 diagnosis codes, age, sex) required for cohort definition and matching. Multiple imputation was therefore not appropriate, as these variables cannot be meaningfully reconstructed. Moreover, available auxiliary variables in statutory health insurance data represent imperfect proxies of clinical and socioeconomic status, such that imputation would introduce model-based assumptions rather than recover valid information and could bias effect estimates. We have clarified this in the revised manuscript.

Change made: Missing data section revised; [MI procedure described / corrected statement about missingness].

Methods: *[…] Minor case-wise deletion was applied because missingness affected core identification variables (ICD-10 diagnosis codes, age, or sex) required for cohort definition and PSM, which cannot be validly imputed without introducing substantial bias.*

Location: Methods—Statistics: p. 9, l. 33; p. 10, l. 1–2;
